# Behavioral Change Towards Reduced Intensity Physical Activity Is Disproportionately Prevalent Among Adults With Serious Health Issues or Self-Perception of High Risk During the UK COVID-19 Lockdown

**DOI:** 10.3389/fpubh.2020.575091

**Published:** 2020-09-30

**Authors:** Nina Trivedy Rogers, Naomi R. Waterlow, Hannah Brindle, Luisa Enria, Rosalind M. Eggo, Shelley Lees, Chrissy h. Roberts

**Affiliations:** ^1^University College London (UCL) Research Department of Epidemiology & Public Health, London, United Kingdom; ^2^London School of Hygiene and Tropical Medicine, London, United Kingdom; ^3^Department of Social & Policy Sciences, University of Bath, Bath, United Kingdom

**Keywords:** physical activity (exercise), COVID-19, SARS-CoV2, lockdown, chronic condition, mixed method approach, perceptions of risk, obesity

## Abstract

**Objectives:** We assessed whether lockdown had a disproportionate impact on physical activity behavior in groups who were, or who perceived themselves to be, at heightened risk from COVID-19.

**Methods:** Physical activity intensity (none, mild, moderate, or vigorous) before and during the UK COVID-19 lockdown was self-reported by 9,190 adults between 2020-04-06 and 2020-04-22. Physician-diagnosed health conditions and topic composition of open-ended text on participants' coping strategies were tested for associations with changes in physical activity.

**Results:** Most (63.9%) participants maintained their normal physical activity intensity during lockdown, 25.0% changed toward less intensive activity and 11.1% were doing more. Doing less intensive physical activity was associated with obesity (OR 1.25, 95% CI 1.08–1.42), hypertension (OR 1.25, 1.10–1.40), lung disease (OR 1.23, 1.08–1.38), depression (OR 2.05, 1.89–2.21), and disability (OR 2.13, 1.87–2.39). Being female (OR 1.25, 1.12–1.38), living alone (OR 1.20, 1.05–1.34), or without access to a garden (OR 1.74, 1.56–1.91) were also associated with doing less intensive physical activity, but being in the highest income group (OR 1.73, 1.37–2.09) or having school-age children (OR 1.29, 1.10–1.49) were associated with doing more. Younger adults were more likely to change their PA behavior compared to older adults. Structural topic modeling of narratives on coping strategies revealed associations between changes in physical activity and perceptions of personal or familial risks at work or at home.

**Conclusions:** Policies on maintaining or improving physical activity intensity during lockdowns should consider (1) vulnerable groups of adults including those with chronic diseases or self-perceptions of being at risk and (2) the importance of access to green or open spaces in which to exercise.

## Introduction

The pandemic spread of Severe Acute Respiratory Syndrome Coronavirus 2 (SARS-CoV-2) (1) was declared a Public Health Emergency of International Concern by the World Health Organization on 30 January 2020^1^ and by the end of April 2020 the virus had infected more than 3 million people worldwide, causing more than 200,000 deaths^2^. In order to limit the spread of Coronavirus disease 2019 (COVID-19), governments across the globe imposed varying degrees of social distancing advice and nationwide lockdowns. On 23 March 2020 the UK government enacted measures that were included in the Coronavirus Act 2020 and recommended that everyone must stay in their homes unless (i) shopping for essentials such as food and medicine, (ii) requiring medical assistance, (iii) caring for vulnerable people, (iv) traveling to and from work if absolutely necessary and (v) to carry out one form of exercise (e.g. walking, running, cycling) each day, either alone or with people who live together. Some adults aged 70 and over and those with specific underlying health conditions including asthma, heart disease, diabetes, and being seriously overweight were also advised to follow stricter social isolation recommendations. In this paper we refer to the combined package of measures as “lockdown”.

There have been growing concerns that the lockdown has placed limitations on opportunities for individuals to be physically active^3^ (2). It is well-established that physical activity (PA), a modifiable behavior, is protective against non-communicable diseases (3–5) and that reduced levels of PA may have a negative impact on the control of chronic health problems including metabolic, cardiovascular, musculoskeletal, pulmonary, and psychiatric conditions; all of which are also often better controlled when PA is included as part of the management plan (6).

The tradeoff between protection from COVID-19 and increased risk of inactivity presents already vulnerable populations with a potential “no-win” situation; for instance, where the consequence of protection from acquiring SARS-CoV-2 infection is increased inactivity and associated downstream health impacts. Longer term, it is also possible that changes in PA behaviors could serve to increase the size of the population that is vulnerable to severe complications from COVID-19 in subsequent epidemic waves. Furthermore, a recent study showed that physical inactivity was one lifestyle-related risk factor for severe COVID-19 requiring hospital admission (7). In this study we identify whether the UK's lockdown measures have had disproportionate impacts on PA intensity in groups who are, or who perceive themselves to be at risk of worse outcomes of COVID-19 disease. This study takes the form of a UK-wide survey of adults aged 20 and over.

## Materials and Methods

### Online Survey

Anonymous survey data were collected online between 2020-04-06 and 2020-04-22, roughly mapping to weeks 3–5 of the lockdown in the UK. The survey included 49 questions which covered a broad range of topics including (1) Demographics, (2) Health and Health Behaviors, (3) Adherence to COVID-19 Control measures, (4) Information sources used to learn about COVID-19, (5) Trust in various information sources, government and government decision-making, (6) Rumors and misinformation, (7) Contact & Communication during COVID-19 and (8) Fear and Isolation.

The survey was publicized using a “daisy-chaining” approach in which respondents were asked to share and to encourage onward sharing of the survey's Uniform Resource Locator (URL) among friends & colleagues. The study team directly targeted a number of faith institutions, schools and special interest groups and also used Facebook's premium “Boost Post” feature. A “boosted” post functions as an advert which can be targeted at specific demographics. We boosted details of the survey and it's URL to a target audience of 113,280 Facebook users aged 13 and over and living in England, Wales, Scotland and Northern Ireland. Participants were also provided with URL links to a set of freely available summary reports and analyses which were periodically updated in near-real time.

We used an ODK XForm (https://getodk.github.io/xforms-spec/) deployed on Enketo smart paper (https://enketo.org/) via ODK Aggregate v.2.0.3 (https://github.com/getodk/aggregate). Form level encryption and end-to-end encryption of data transfer were implemented on all submissions.

### Disability and Classification of Health Conditions

Participants were assessed for disability by asking about difficulties in six activities of daily living (ADLs) (8) including bathing, dressing, walking across a room, eating (such as cutting up food), getting in and out of bed, and using the toilet (including getting up and down). Disability was defined by the presence of at least one ADL. We also explored depressive symptomatology with the question “In the past 2 weeks, how often have you felt down, depressed, or hopeless?”. Options were “not at all”, “several days”, “more than half the days”, and “every day”. Participants were classified as currently depressed if they reported feeling this way either “more than half the days” or “every day”. To determine whether patients had any previous or current chronic disease(s) diagnosis (CDD), participants were asked “Has a doctor ever diagnosed you with any of the following?”. The question allowed for multiple chronic diseases to be selected from a list that included diabetes type 1, diabetes type 2, lung disease, cancer, stroke, heart disease, high blood pressure (hypertension) and obesity.

We additionally asked participants to provide (in narrative text form) details about any other medical conditions that they felt would increase their risk of getting seriously ill if they were to catch coronavirus. We chose to recode any participant who mentioned asthma as having a lung disease because the topic of “Asthma” accounted for around 25% of the open text responses to this question (Determined by structural text modeling, see below) and because asthma was mentioned directly by 678 participants (Supplementary Figure 1). The majority of people who reported having a doctor's diagnosis of lung disease also mentioned asthma (63.4%, *n* = 225, Supplementary Table 1) suggesting that they operationalized asthma as a lung disease and may have been referring to asthma when they reported their prior diagnosis of lung disease. 8.3% (*n* = 453) of people who did not report having “lung disease” did however mention asthma in the free text.

### Analysis

We handled missing data by performing multiple imputation by chained equations using the *mice* package for R and 20 imputed data sets were completed for all relevant fields including highest educational qualifications, age (20–34; 35–54; 55–69; 70+), gender, whether living alone, household income, presence of ADLs, self-rated depression and pre-existing chronic diseases. Pearson's χ^2^ test was used to detect factor variables with statistically significant differences between the groups when the data were grouped according to baseline (pre-COVID-19) PA levels. Due to significant differences according to baseline PA, all further analyses were corrected for baseline PA. The main response variable for statistical association tests was any change in PA intensity from pre-COVID-19 lockdown to the time of survey participation. This value was calculated by comparing baseline PA (“Before the outbreak began, what type of exercise did you regularly do?”, options “None”, “Mild [e.g. walking short distances, doing DIY etc.]”, “Moderate [e.g., A gentle workout, Digging the garden, Dancing]”, & “Vigorous [e.g., Running/Jogging/Hiking, Cycling, Weightlifting]”) to PA during COVID-19 lockdown (“What type of exercise are you doing now?”, options as for baseline). Participants were classified as doing the “Same”, “Less”, or “More” than their usual PA intensity. In order to detect factors which were associated with change in PA intensity during lockdown we used the “nnet” R package applied to a multinomial log-linear model via neural networks (9) to the imputed data set, pooling the results of the 20 separate analyses using Rubin's rules.

### Topic Modeling

We used Structural Text Modeling (STM) (10) to identify key topics in the data on self-perceived medical risk factors (see above) and also to determine whether changes in PA intensity were associated with participants' other perceptions of risk from COVID-19. STM employs machine learning (ML) approaches to explore open ended survey questions in a highly structured and reproducible way (10). The goal of STM is to identify topics and perspectives in free-text data, for instance by highlighting specific diseases, themes or perspectives being reported in the survey. This is functionally analogous and equivalent in results to the type of human coding of text data performed by anthropologists and ethnographers; but unlike more conventional topic modeling, STM makes it possible to link topic models to metadata and quantitative data in a way that is directly amenable to statistical modeling (10, 11). All STM was performed using the “stm” package for R (11). STM was applied to data from the open-ended survey question “On 23rd March 2020, the Prime Minister Boris Johnson announced a complete lockdown in the UK. Tell us what you have been doing to help you cope during this difficult time?”. The text data were processed into a corpus and transliterated to lower-case. Numbers, common punctuation and stop-words (such as “I”, “me”, “that's”, and “because”) were stripped and data were trimmed to include only words which appeared in 20 or more responses to the survey. The corpus was then bound to the quantitative data from the survey and the STM was optimized to determine the number of topics which maintained the balance between high semantic coherence (i.e. the topics were clear and understandable) and exclusivity (vocabulary and themes had little cross-over between topics). The topics were then labeled manually (this and defining the number of topics of interest were the only subjective components of the process) by first examining the word usage within topics (weighted by exclusivity) and then assessing a number of representative perspectives (quotes) from each of the topics. Expected text proportions (ETP) were defined as the proportion of the total corpus which related to each topic. Between-topic correlations were measured using the semiparametric procedure described in the R package “huge” (High-Dimensional Undirected Graph Estimation). Tests for statistical associations between the PA data set and the STM topics used regressions of the STM, where the between group ETPs were the outcome variables and the survey PA question data, including the change in PA intensity were the explanatory variables.

### Patient and Public Involvement

This study is a collaboration between two continuing National Institute of Health Research (NIHR) funded programmes of research including (i) The Emergency and Epidemic Data Kit [EDK] and (ii) Anthropological Exploration of Facilitators and Barriers to Vaccine Deployment and Administration During Disease Outbreaks (AViD). Both projects have been developed and guided from the earliest stages by patient and public involvement and stakeholders have been included in all stages of the research. The open source survey software used in this study was developed in collaboration with a global community of researchers, data scientists and field epidemiologists, including members of the public, not-for-profit organizations and partners from low and middle income countries. A group of around 15 lay members of the UK public, including both younger and older people, were asked to review and recommend changes to the content of the survey before it was fully deployed.

## Results

The survey consisted of 9,456 participants. The majority of respondents (78%) were female and most (82%) were aged between 35 and 69 years. There was a relatively normal distribution of household incomes but a large proportion of the participants (62%, *n* = 5,502) were educated to degree level or higher. Participants lived across the UK including 6% in Scotland, 5% in Wales, 1% in Northern Ireland and of those from England, 35% in London and the South-East regions. Ethnically, 95% of participants were white, with just 3.7% being from black and minority ethnic (BAME) backgrounds. 0.9% of respondents opted not to reveal their ethnicity. Ethnicities were not included as a covariate in statistical analyses as the numbers in each group were too small. Participants who were transgender, gender fluid, or non-binary, and those who indicated that they did not wish to reveal their gender (*n* = 73) were grouped together under the term “all other genders” for statistical reasons, although we acknowledge that this grouping does not fully recognize the individuality, granularity and diversity of the gender identities of our participants. The prevalence of adults carrying out moderate or vigorous PA on a regular basis was similar to that previously reported for adults living in England^4^.

After filtering the data (Supplementary Table 2) we retained 9,190 participants for analysis and demographic characteristics of the filtered sample are given in Table 1. At least one data point was missing and therefore imputed in 3,294 (35.8%) responses. The percentages (and number) of imputed data points were as follows: education (2.6%, *n* = 237), gender (0.7%, *n* = 60), access to garden (0.3 %, *n* = 23), school age children (0.7%, *n* = 67), income (13.2%, *n* = 1,214), depression (0.8%, *n* = 69), diabetes type I (18.2%, *n* = 1,671), diabetes type II (15.5%, *n* = 1,429), lung disease (15.5%, *n* = 1,426), cancer (17.5%, *n* = 1,610), stroke (20.0%, *n* = 1,840), heart disease (18.5%, n = 1,704), hypertension (9.6%, *n* = 879), and obesity (14.7%, *n* = 1,353). Full details of the number of observations contributing to the multinomial regression analysis, including a breakdown of observed and imputed numbers of observations by change in PA intensity are provided in Supplementary Table 3.

**Table 1:** Demographic characteristics of the sample, by baseline PA intensity.

		**PA Intensity before lockdown**^****a****^					
**Variable**	**Group**	**None**	**Mild**	**Moderate**	**Vigorous**	**Total^**b**^**	***p*-value^**c**^**
		**(*N* = 288)**	**(*N* = 3,111)**	**(*N* = 3,874)**	**(*N* = 1,917)**	**(*N* = 9,190)**	
PA intensity during lockdown *n* (%)	None	172 (23.2%)	390 (52.7%)	132 (17.8%)	46 (6.2%)	740 (8.05 %)	<0.001
	Mild	76 (2.3%)	1,999 (60.5%)	977 (29.6%)	252 (7.6%)	3,304 (35.95 %)	
	Moderate	31 (0.8%)	641 (17.0%)	2,587 (68.7%)	505 (13.4%)	3,764 (40.96 %)	
	Vigorous	9 (0.7%)	81 (5.9%)	178 (12.9%)	1,114 (80.6%)	1,382 (15.04 %)	
PA Change during lockdown *n* (%)	Same	172 (2.9%)	1,999 (34.0%)	2,587 (44.1%)	1,114 (19.0%)	5,872 (63.9 %)	<0.001
	Less	0 (0.0%)	390 (16.9%)	1,109 (48.2%)	803 (34.9%)	2,302 (25.05 %)	
	More	116 (11.4%)	722 (71.1%)	178 (17.5%)	0 (0.0%)	1,016 (11.06 %)	
Age *n* (%)	20-34	21 (3.4%)	157 (25.4%)	195 (31.6%)	245 (39.6%)	618 (6.72 %)	<0.001
	35-54	116 (3.5%)	1,060 (32.4%)	1,221 (37.3%)	877 (26.8%)	3,274 (35.63 %)	
	55-69	126 (3.0%)	1,456 (34.4%)	1,952 (46.1%)	702 (16.6%)	4,236 (46.09 %)	
	70+	25 (2.4%)	438 (41.2%)	506 (47.6%)	93 (8.8%)	1,062 (11.56 %)	
Gender *n* (%)	Female	224 (3.1%)	2,474 (34.6%)	3,146 (44.0%)	1,299 (18.2%)	7,143 (77.73 %)	<0.001
	Male	55 (2.9%)	598 (31.2%)	675 (35.3%)	586 (30.6%)	1,914 (20.83 %)	
	All other genders	8 (11.0%)	21 (28.8%)	22 (30.1%)	22 (30.1%)	73 (0.79 %)	
Living alone *n* (%)	No	226 (3.0%)	2530 (33.0%)	3246 (42.4%)	1659 (21.7%)	7,661 (83.36 %)	<0.001
	Yes	62 (4.1%)	581 (38.0%)	628 (41.1%)	258 (16.9%)	1,529 (16.64 %)	
Education *n* (%)	GCSE/O-level	47 (5.1%)	399 (43.0%)	377 (40.6%)	105 (11.3%)	928 (10.1 %)	<0.001
	A level/Highers	86 (3.4%)	963 (38.2%)	1,089 (43.2%)	385 (15.3%)	2,523 (27.45 %)	
	Degree	143 (2.6%)	1640 (29.8%)	2,314 (42.1%)	1,405 (25.5%)	5,502 (59.87 %)	
Access to a garden *n* (%)	Yes	249 (3.0%)	2,792 (33.5%)	3,593 (43.1%)	1,699 (20.4%)	8,333 (90.67 %)	<0.001
	No	36 (4.3%)	310 (37.2%)	271 (32.5%)	217 (26.0%)	834 (9.08 %)	
School aged children *n* (%)	No	220 (3.1%)	2,493 (34.8%)	3,088 (43.1%)	1367 (19.1%)	7,168 (78 %)	<0.001
	Yes	67 (3.4%)	594 (30.4%)	755 (38.6%)	539 (27.6%)	1,955 (21.27 %)	
Household Income *n* (%)	< £15,000	59 (5.7%)	455 (43.8%)	420 (40.4%)	105 (10.1%)	1,039 (11.31 %)	<0.001
	£15,000–£24,999	36 (2.4%)	583 (38.8%)	669 (44.5%)	215 (14.3%)	1,503 (16.35 %)	
	£25,000–£39,999	49 (2.7%)	641 (35.1%)	783 (42.9%)	351 (19.2%)	1,824 (19.85 %)	
	£40,000–£59,999	57 (3.4%)	491 (29.5%)	698 (42.0%)	417 (25.1%)	1,663 (18.1 %)	
	£60,000–£99,999	27 (2.0%)	376 (28.3%)	526 (39.5%)	401 (30.2%)	1,330 (14.47 %)	
	More than £100,000	12 (1.9%)	154 (25.0%)	220 (35.7%)	231 (37.4%)	617 (6.71 %)	
Disability (ADL) *n* (%)	No	208 (2.4%)	2,879 (32.7%)	3,808 (43.3%)	1,897 (21.6%)	8,792 (95.67 %)	<0.001
	Yes	80 (20.1%)	232 (58.3%)	66 (16.6%)	20 (5.0%)	398 (4.33 %)	
Depression *n* (%)	No	238 (2.9%)	2,746 (33.4%)	3,516 (42.8%)	1,716 (20.9%)	8,216 (89.4 %)	<0.001
	Yes	48 (5.3%)	344 (38.0%)	328 (36.2%)	185 (20.4%)	905 (9.85 %)	
Diabetes type I *n* (%)	No	222 (3.0%)	2,400 (32.3%)	3,126 (42.0%)	1,689 (22.7%)	7,437 (80.92 %)	0.014
	Yes	5 (6.1%)	37 (45.1%)	23 (28.0%)	17 (20.7%)	82 (0.89 %)	
Diabetes type II *n* (%)	No	201 (2.8%)	2,264 (31.5%)	3,049 (42.5%)	1668 (23.2%)	7,182 (78.15 %)	<0.001
	Yes	46 (7.9%)	302 (52.2%)	188 (32.5%)	43 (7.4%)	579 (6.3 %)	
Lung Disease *n* (%)	No	179 (2.8%)	1,996 (31.5%)	2,678 (42.3%)	1485 (23.4%)	6,338 (68.97 %)	<0.001
	Yes	57 (4.0%)	553 (38.8%)	573 (40.2%)	243 (17.0%)	1,426 (15.52 %)	
Cancer *n* (%)	No	201 (2.9%)	2,206 (32.1%)	2,880 (41.9%)	1,593 (23.2%)	6,880 (74.86 %)	0.026
	Yes	24 (3.4%)	247 (35.3%)	324 (46.3%)	105 (15.0%)	700 (7.62 %)	
Stroke *n* (%)	No	202 (2.8%)	2,305 (32.0%)	3,033 (42.1%)	1,656 (23.0%)	7,196 (78.3 %)	<0.001
	Yes	15 (9.7%)	62 (40.3%)	58 (37.7%)	19 (12.3%)	154 (1.68 %)	
Heart disease *n* (%)	No	201 (2.8%)	2,250 (31.8%)	2,990 (42.3%)	1,630 (23.1%)	7,071 (76.94 %)	<0.001
	Yes	28 (6.7%)	186 (44.8%)	150 (36.1%)	51 (12.3%)	415 (4.52 %)	
Hypertension *n* (%)	No	162 (2.6%)	1,817 (29.6%)	2,639 (43.0%)	1,523 (24.8%)	6,141 (66.82 %)	<0.001
	Yes	94 (4.3%)	952 (43.9%)	866 (39.9%)	258 (11.9%)	2,170 (23.61 %)	
Obesity *n* (%)	No	145 (2.3%)	1,811 (28.7%)	2,771 (44.0%)	1,577 (25.0%)	6,304 (68.6 %)	<0.001
	Yes	105 (6.8%)	803 (52.4%)	480 (31.3%)	145 (9.5%)	1,533 (16.68 %)	

a PA Intensity before lockdown shows percentage of each class doing different PA intensity before lockdown.

b Total shows percentage of each class in the total sample of 9,190 participants.

c *P-value: Pearson's Chi Squared Test*.

All statistical testing used the group who had not changed PA intensity as the reference group.

**Figure 1:**
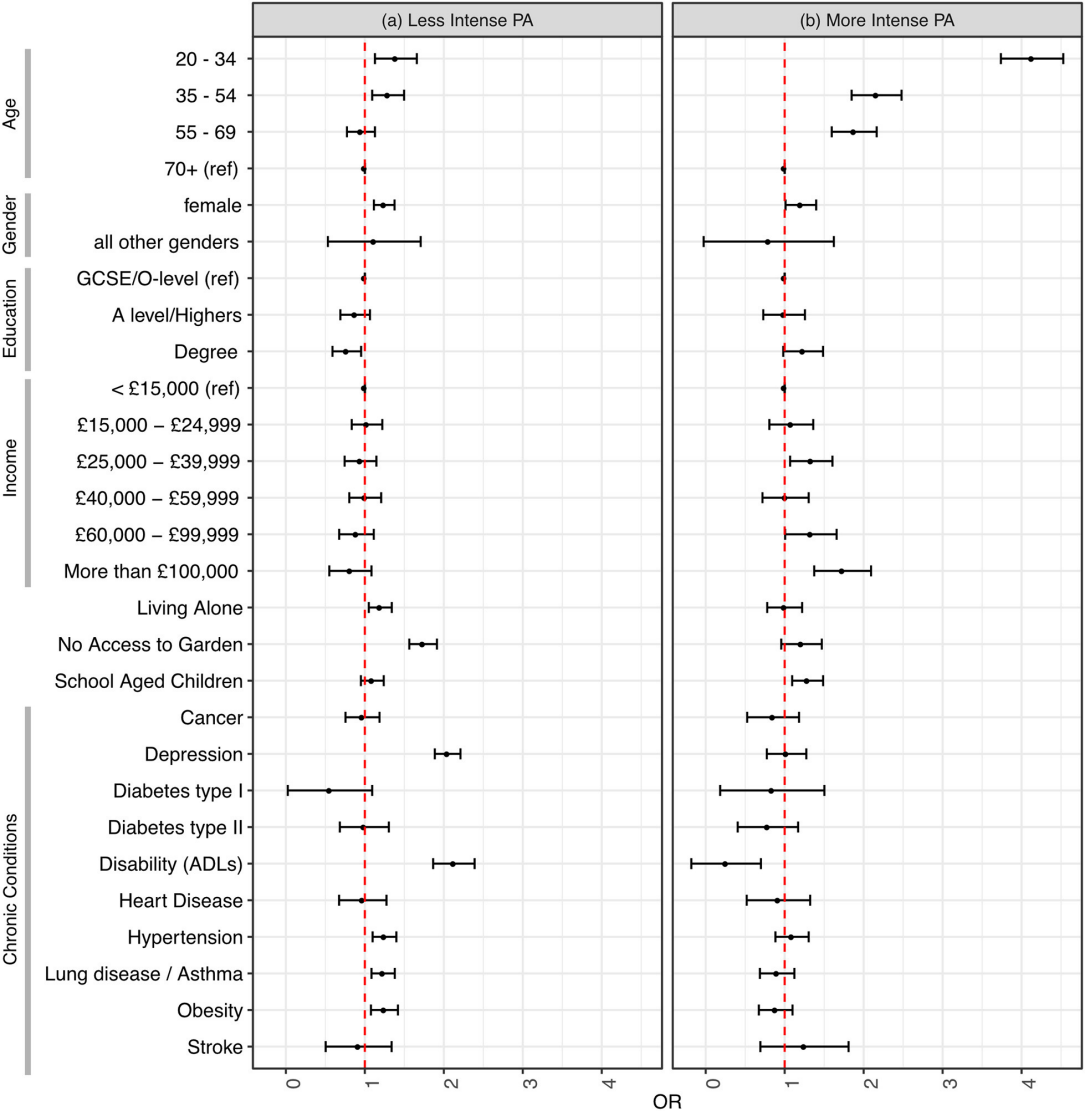
Odds ratios for having changed toward **(a)** less intense and **(b)** more intense physical activity since the UK COVID-19 lockdown began. The reference group is study participants who continued to do the same intensity of physical activity. All odds ratios are corrected for baseline physical activity intensity and all other variables.

Approximately 36% of participants (*n* = 3,318) reported a change in their PA behaviors during lockdown, with 25.1% (*n* = 2,302) doing less and 11.1% (*n* = 1,016) doing more than before the pandemic. After correcting for baseline PA intensity, there were significantly increased odds for women (compared to men) to have started doing less intense PA under lockdown (OR 1.25, 95% CI 1.12–1.38, *p* = 0.001). This was also the case for people who did not have access to a garden (OR 1.74, 95% CI 1.56–1.91, *p* < 0.001) or whom lived alone (OR 1.20, 95% CI 1.05–1.34, *p* = 0.016). Decreasing age had a linear relationship to the odds of changing PA behaviors in either direction (Figure 1, Table 2). Lung diseases were significantly associated with increased odds of change toward doing less intense PA (OR 1.23, 95% CI 1.08–1.38, *p* = 0.006). Hypertension (OR 1.25, 95% CI 1.10–1.40, *p* = 0.004), depression (OR 2.05, 95% CI 1.89–2.21, *p* < 0.001) and disability from one or more ADL (OR 2.13, 95% CI 1.87–2.39, *p* < 0.001) were all significantly associated with change toward less intense PA behaviors (Table 2, Figure 1). Participants from the wealthiest group (OR 1.73, 95% CI 1.37–2.09, *p* = 0.003) and those living with school aged children (OR 1.29, 95% CI 1.10-1.49, p = 0.011) had increased odds of changing to do more intense PA compared to, respectively, the least wealthy group and those living without children of school age.

**Table 2:** Odds ratios for change in intensity of physical activity toward (a) less intense and (b) more intense physical activity since the UK COVID-19 lockdown began.

		**Corrected for age and sex**		**Fully Corrected**^****a****^	
**Variable**	**Group**	**OR^**b**^**	***P***	**OR^**b**^**	***P***
**(A) LESS INTENSE PHYSICAL ACTIVITY**					
Age	20–34	1.47 (1.23–1.70)	0.002	1.39 (1.13–1.66)	0.014
	35–54	1.24 (1.06–1.41)	0.016	1.30 (1.09–1.50)	0.012
	55–69	0.93 (0.76–1.10)	0.395	0.95 (0.77–1.13)	0.575
	70 +	REF	-	REF	-
Gender	Female	1.27 (1.15–1.40)	<0.001	1.25 (1.12–1.38)	0.001
	All other genders	1.41 (0.85–1.96)	0.232	1.12 (0.53–1.71)	0.707
	Male	REF	-	REF	-
Education	GCSE/O-level	REF	-	REF	-
	A level/Highers	0.89 (0.70–1.07)	0.194	0.88 (0.69–1.07)	0.172
	Degree	0.74 (0.57–0.91)	0.001	0.77 (0.59–0.95)	0.005
Income	< £15,000	REF	-	REF	-
	£15,000-£24,999	0.90 (0.71–1.09)	0.277	1.03 (0.83–1.22)	0.781
	£25000-£39,999	0.78 (0.59–0.97)	0.011	0.95 (0.74–1.15)	0.581
	£40,000-£59,999	0.79 (0.60–0.98)	0.013	1.01 (0.80–1.21)	0.961
	£60,000-£99,999	0.67 (0.46–0.87)	<0.001	0.89 (0.68–1.11)	0.317
	> £100,000	0.60 (0.35–0.85)	<0.001	0.82 (0.55–1.08)	0.139
Living alone	Yes	1.35 (1.22–1.49)	<0.001	1.20 (1.05–1.34)	0.016
Access to garden	No	1.86 (1.70–2.03)	<0.001	1.74 (1.56–1.91)	<0.001
School Aged Children	Yes	0.95 (0.82–1.09)	0.507	1.09 (0.95–1.24)	0.222
Health conditions	Cancer	1.00 (0.83–1.16)	0.977	0.97 (0.76–1.19)	0.787
	Depression	2.22 (2.07–2.38)	<0.001	2.05 (1.89–2.21)	<0.001
	Diabetes type I	0.84 (0.54–1.14)	0.263	0.56 (0.02–1.09)	0.035
	Diabetes type II	1.13 (0.95–1.32)	0.195	0.99 (0.68–1.30)	0.967
	Disability (ADLs)	2.54 (2.29–2.80)	<0.001	2.13 (1.87–2.39)	<0.001
	Heart Disease	1.02 (0.83–1.22)	0.835	0.97 (0.67–1.27)	0.861
	Hypertension	1.29 (1.17–1.42)	<0.001	1.25 (1.10–1.40)	0.004
	Lung disease/Asthma	1.25 (1.12–1.38)	0.001	1.23 (1.08–1.38)	0.006
	Obesity	1.33 (1.20–1.47)	<0.001	1.25 (1.08–1.42)	0.012
	Stroke	0.98 (0.72–1.23)	0.855	0.92 (0.50–1.34)	0.701
**(B) MORE INTENSE PHYSICAL ACTIVITY**					
Age	20-34	5.60 (5.24–5.96)	<0.001	4.13 (3.74–4.53)	<0.001
	35-54	2.89 (2.61–3.17)	<0.001	2.16 (1.85–2.48)	<0.001
	55-69	1.95 (1.67–2.22)	<0.001	1.88 (1.60–2.17)	<0.001
	70 +	REF	-	REF	-
Gender	Female	1.12 (0.93–1.30)	0.251	1.21 (1.01–1.40)	0.059
	All other genders	0.89 (0.08–1.69)	0.765	0.80 (-0.03–1.62)	0.593
	Male	REF	-	REF	-
Education	GCSE/O-level	REF	-	REF	-
	A level/Highers	1.05 (0.79–1.30)	0.742	0.99 (0.73–1.26)	0.960
	Degree	1.43 (1.19–1.68)	0.003	1.23 (0.98–1.49)	0.103
Income	< £15,000	REF	-	REF	-
	£15,000-£24,999	1.18 (0.91–1.44)	0.237	1.08 (0.81–1.36)	0.570
	£25,000-£39,999	1.52 (1.26–1.77)	0.001	1.34 (1.07–1.61)	0.034
	£40,000-£59,999	1.24 (0.96–1.51)	0.127	1.01 (0.72–1.30)	0.938
	£60,000-£99,999	1.68 (1.38–1.98)	0.001	1.33 (1.01–1.66)	0.086
	> £100,000	2.26 (1.92–2.59)	<0.001	1.73 (1.37–2.09)	0.003
Living alone	Yes	0.89 (0.69–1.09)	0.233	1.00 (0.78–1.22)	0.999
Access to garden	No	1.11 (0.86–1.35)	0.418	1.21 (0.96–1.47)	0.14
School aged children	Yes	1.36 (1.18–1.55)	0.001	1.29 (1.10–1.49)	0.011
Health conditions	Cancer	0.69 (0.44–0.94)	0.004	0.85 (0.53–1.18)	0.346
	Depression	0.87 (0.63–1.11)	0.260	1.02 (0.77–1.27)	0.853
	Diabetes type I	0.57 (0.16–0.98)	0.009	0.84 (0.18–1.50)	0.612
	Diabetes type II	0.60 (0.33–0.86)	<0.001	0.79 (0.41–1.17)	0.221
	Disability (ADLs)	0.22 (–0.21–0.66)	<0.001	0.26 (–0.18–0.70)	<0.001
	Heart Disease	0.64 (0.36–0.92)	0.002	0.92 (0.52–1.32)	0.688
	Hypertension	0.81 (0.64–0.99)	0.020	1.09 (0.88–1.30)	0.405
	Lung disease/Asthma	0.73 (0.53–0.92)	0.001	0.91 (0.69–1.12)	0.370
	Obesity	0.73 (0.56–0.91)	0.001	0.89 (0.67–1.10)	0.265
	Stroke	0.63 (0.28–0.99)	0.012	1.25 (0.69–1.81)	0.433

a Fully corrected model was adjusted for baseline physical activity intensity and all other variables.

b *All odds ratios are compared to the baseline group of those who maintained their physical activity intensity*.

**Figure 2:**
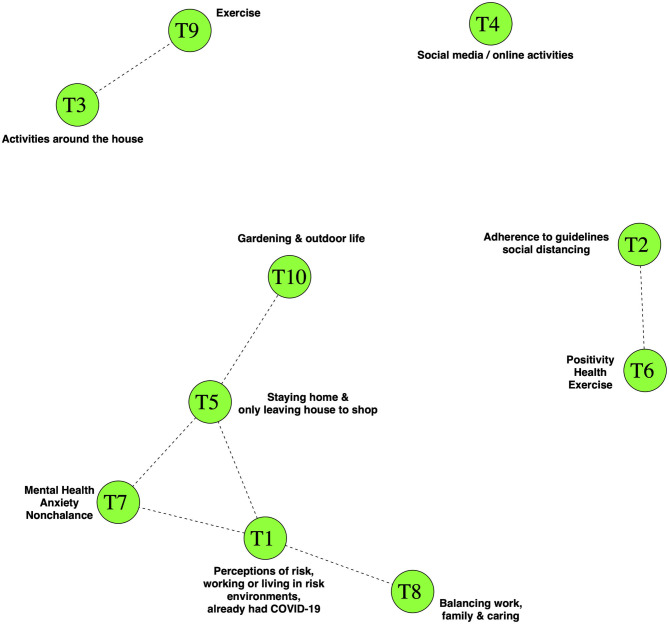
Topic Correlations in the structural topic model for the corpus of text describing coping strategies. Nodes show topics and lines show positive correlations between the topics. There was a close correlation between topic T1 “Perceptions of risk, working or living in risk environments, already had COVID-19” and topics relating to mental health & anxiety (topic T7), strict adherence to social isolation practices (topic T5) and the balancing of ongoing work and family commitments (topic T8). Participants whose responses were focussed toward discussion of more intense PA (topic T9) or activities (including PA) around the house (topic T3) were less likely to also focus on T1, T7, T8, or T5. The topic correlation cut-off was 0.01.

To investigate the role of self-perceived risks on PA behaviors during lockdown, we used STM to reveal 10 topics in the 8,642 survey responses which constituted the corpus of text on the coping-strategies of the study participants. The 10 key topics we identified were (T1) “Perceptions of risk/working or living in risk environments/already had COVID-19”, (T2) “Adherence to guidelines/social distancing”, (T3) “Activities around the house”, (T4) “Social media/online activities”, (T5) “Staying home & only leaving house to shop”, (T6) “Positivity/ Health/Exercise”, (T7) “Mental Health/Anxiety/Nonchalance”, (T8) “Balancing work, family and caring”, (T9) “Exercise”, and (T10) “Gardening & outdoor life”. Representative perspectives (in the form of quotes) from the topics are provided in Supplementary Table 4. Some of these topics were correlated (Figure 2), suggesting that participant narratives may have had thematic overlaps between correlated topics. The correlation between Topics T3 “Activities around the house” and T9 “Exercise” suggest that a substantial proportion of participants who discussed doing exercises like yoga, running or walking also discussed keeping busy around the house with tasks such as cooking, knitting, gardening and reading, or doing jigsaws. Those whose responses included T6 “Positivity/Health/Exercise”, a topic which included references to maintaining routines, keeping in touch with friends and staying active, potentially also talked about T2 “Adherence to guidelines/social distancing”.

Topic T1 “Perceptions of risk…” included quotes which referenced working in high risk settings such as the national health service and also references to living with vulnerable family members. A number of quotes from T1 also came from individuals who claimed to have had suffered from COVID-19 either previously or at the time of participation in the study. Topic T1 appeared to correlate closely with T8 “balancing work, family and caring” and with T7 which included comments about the effect of the pandemic on the mental health of the participants. T7 included both very anxious and surprisingly nonchalant or fatalistic comments. Topics T1 and T7 were both in turn correlated with Topic T5, which described how some participants had stayed home entirely, leaving only to shop for necessities.

**Figure 3:**
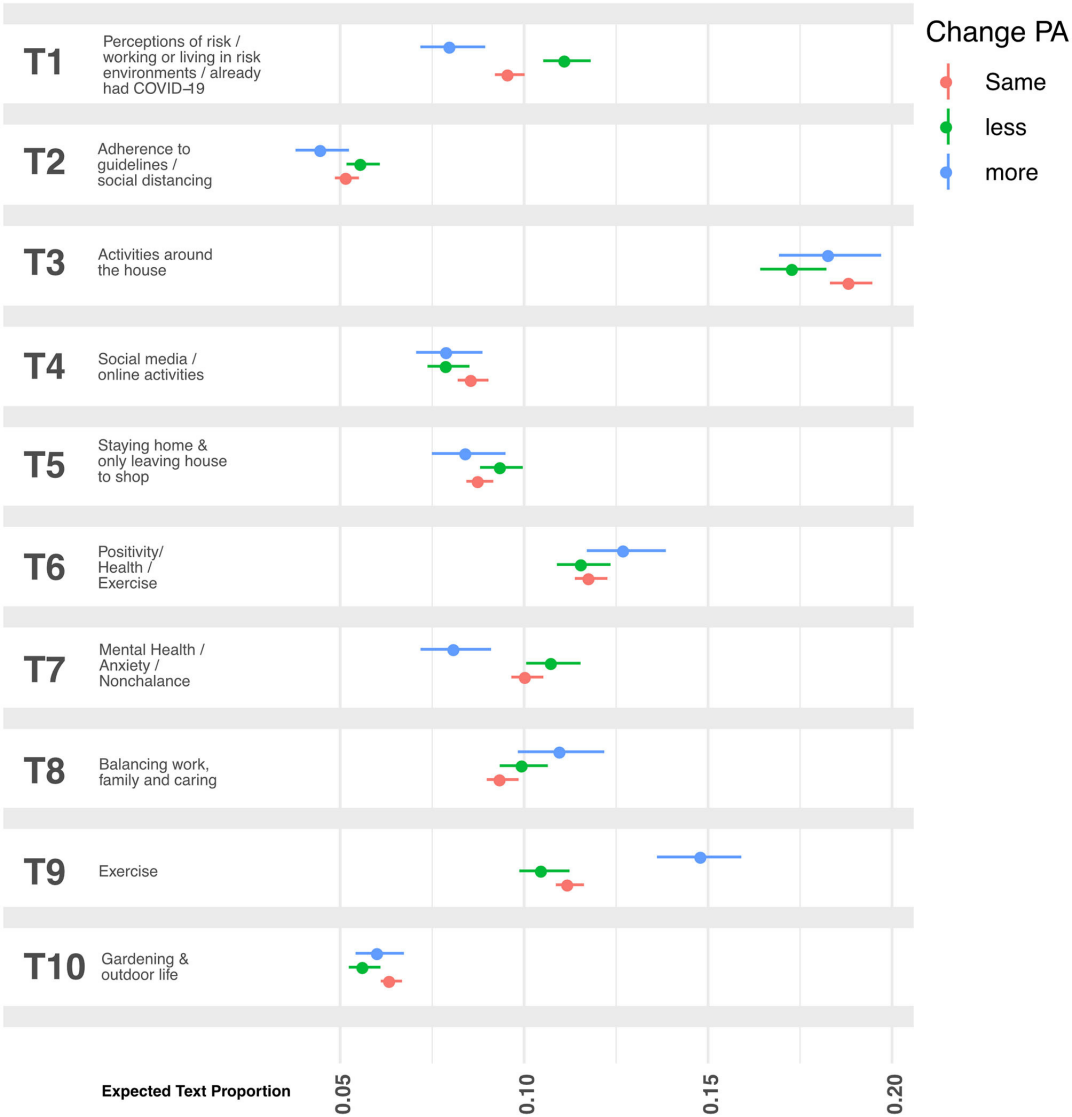
Expected text proportions in open-ended narratives on participants' coping behaviors. Participants were asked to describe their coping behaviors during the UK COVID-19 related lockdown. Perceptions of risk, working or living in risk environments, and already having had COVID-19 (topic T1) were mentioned in 9.7% (9.3–10.1%) of responses from participants who had not changed, in 8.1% (7.1–9.0%, *p* = 0.001) of responses from people doing more PA and in 11.2% (10.4–11.9%, *p* < 0.001) among participants doing less PA. Topic T3 (*p* = 0.01) and T10 (*p* = 0.006) had lower ETPs in the PA group doing “less”. Topics T7 and T8 had, respectively, lower (*p* < 0.001) and higher (*p* = 0.006) in the PA group doing “more”. Topic T9 “Exercise” was notably much higher in the group which reported doing more intense PA under lockdown (*p* < 0.001). All analysis used the group which had not changed PA intensity as the reference.

When we performed a statistical analysis of how change in PA intensity related to coping strategies during lockdown, the STM expected text proportions revealed that “Perceptions of risk…” (topic T1) were mentioned in 9.7% (9.3–10.1%) of responses from participants who had not changed, in 8.1% (7.1–9.0%, *p* = 0.001) of responses from people doing more PA and in 11.2% (10.4–11.9%, *p* < 0.001) among participants doing less PA (Figure 3). Compared to the ETPs of the group which maintained the same level of PA intensity, topics T3 “Activities around the house” (*p* = 0.01) and T10 “Gardening and outdoor life” (*p* = 0.006) had lower ETPs among those doing less intense PA during lockdown. Topic T7 “Mental health…” had a lower ETP (*p* < 0.001) among those doing more intense PA, whilst the ETP of T8 “Balancing work, family and caring” was higher among this group (*p* = 0.006). Topic T9 “Exercise” was notably much higher in the group which reported doing more intense PA under lockdown (*p* < 0.001).

## Discussion

In this large UK-wide survey of adults aged 20 and over we show that the majority (>60%) of the study sample succeeded in maintaining their normal PA intensity level during the study period of COVID-19 lockdown. Among those who changed their PA levels, more than twice as many people reduced their PA intensity as increased it. Adults who reported having a doctor's diagnosis of obesity, hypertension, lung disease (including asthma) or who indicated depression or disability were more likely to be doing less intensive PA compared to their activity before the epidemic. Compared to the oldest age group (70+), younger age groups were significantly more likely to have changed and to be doing either more, or indeed less intense PA since the lockdown began. Being female, living alone or being without a garden were also associated with doing less intensive PA during the study period, but having school-aged children was associated with doing more. Importantly, we found these associations were independent from all identified confounders.

Analysis of open-ended text data about participants' lockdown coping strategies revealed that people who expressed sentiments about personal, work-related or household risks were more likely to have exhibited a PA behavior change toward less intense activity. This is important because subjective perceptions of risk may act as a conditioning factor that influences decision-making and behavioral change. The strengths of this study include the large population sample of adults who provided information on a wide range of demographic factors and health conditions in addition to PA behaviors before and during the COVID-19 lockdown. Our mixed methods approach allowed us to capture not only objective medical risks for COVID-19 from doctor diagnosed conditions, but also participants' self-perceived risks which make less intensive PA more likely. We applied a recently described ML approach to the codification of topics from open-ended questions, eliminating much of the subjectivity that is usually associated with anthropological & ethnographic approaches to text-mining.

Limitations of the study do exist, in particular because the study findings are not generalizable. The sampling approach was non-random, meaning that the sample was unlikely to fully reflect the demographics of the UK population. As with many epidemiological surveys, participants were disproportionately likely to be highly educated, white and female. The study relied on self-reported information (e.g. intensity of PA, medical conditions) leaving it susceptible to response bias (e.g. imprecise recall, influence of social desirability), however we minimized this where possible, for instance by giving examples of different types of physical activity, with corresponding intensities and asking about medical conditions that had been diagnosed by a physician. Whilst the ML approach we used for text mining was fully reproducible and largely autonomous, topic labels were added manually and the findings of this part of the work should be interpreted with reference to the perspectives presented in Supplementary Table 4. This study is observational and therefore causal links between the outcomes and exposures cannot be assumed. Confounders which were not included in the study, or any that were misclassified, may lead to residual confounding. A significant limitation is that we could not assess the role of ethnicity, which is particularly important because there is substantial evidence that there is a disproportionate effect of COVID-19 on minority ethnic groups (12, 13) and because people from minority ethnic groups have worse health than the overall population, especially among those over 60 (14).

The extent to which adults in the UK will revert back to their usual PA regimes once lockdown measures are relaxed is unclear, but the potential for multiple lockdowns being necessary over a protracted period could lead to prolonged periods of low PA in a substantial proportion of the population. This is concerning because it is well-established that insufficient levels of PA are associated with poor mental (3) and physical (4, 5) health and with premature mortality (6). Furthermore, a reduction in PA levels for even short durations (e.g. a decrease in step-counts per day for 2 weeks) are associated with indicators of poor health including reduced insulin sensitivity, cardiorespiratory fitness, muscle mass, and increased central fat (15, 16). The results of our current study suggest that the health of adults who have disabilities, depression, obesity, hypertension and lung disease may be disproportionately impacted because they are more likely to reduce the intensity of their PA during periods of lockdown. These findings are important because PA is therapeutic in the management of many diseases (6). For example, we observed that adults with disabilities had more than twice the odds (compared to people without disabilities) of having reduced PA intensity during lockdown. This could lead to increased incidence and progression of disablement in older or diseased populations (17).

Recently published recommendations for self-isolation suggest that during periods of lockdown, individuals should attempt to increase their PA (even if only by a little) and exercise every day in order to improve physical cardio-respiratory fitness in case they were to contract coronavirus and become severely ill.^1^ This advice may be even more pertinent for those who are at higher risk of complications from underlying health conditions such as obesity and lung conditions, or for those without gardens. Our findings suggest that these sub-populations are more likely to be doing less than before the lockdown. New advice that promotes home-based exercises such as including extra daily step counts^1^ and more intensive forms of PA (18) should be considered as part of any new public health guidelines for self-isolation and future lockdowns. Targeting PA health messaging to address the potential harms of subjective risks may also be key, given that those who have little or no known objective clinical risk in the current epidemic may change PA behavior in light of their perception of risk, thereby driving the development of clinical risk factors and as a consequence potentially suffering more severe sequelae of SARS-CoV-2 infections during future epidemics.

During the first phase of UK lockdown, many public parks and gardens, exercise equipment in parks and other open spaces were closed. During this period, exercise outside the home was permitted only once a day and it is possible that this left too little opportunity for many to maintain their normal PA. In this survey we have identified the lack of access to a garden during lockdown as a substantial risk factor for doing less intense PA. This is likely because those without gardens simply had more limited opportunities for PA than those who had access to private outdoor space. In any future lockdown, policy-makers should ensure that public open spaces are kept open and made available for PA use by people who have no access to a garden.

To date, studies examining changes in PA before and during COVID-19 lockdown are limited in number but the results of this study are in line with recent findings from an online survey (n = 1,047) of participants from across different continents, which indicate that home confinement due to COVID-19 could negatively impact participation in PA such that it was associated with a 35% reduction (equivalent to 2.45 days) in the number of days per week walking (19).

We conclude that Lockdown measures due to COVID-19 were associated with a change in PA intensity in 37% of study participants. Reduction in the intensity of PA was common and adults with obesity, hypertension, lung disease, disability and depression had increased odds of doing less intense PA than other groups. Participants more frequently expressed sentiments and perspectives on risk when they had changed toward less intense PA. Future research questions and policy should examine how adults with existing chronic health conditions or perceptions of risk can maintain a healthy PA regime (taking in to account the role of accessible outdoor spaces) whilst being confident that they are not, by doing so, placing themselves, their family or their community at increased risk from SARS-CoV2.

## Data Availability Statement

The datasets presented in this study can be found in online repositories. The names of the repository/repositories and accession number(s) can be found below: LSHTM Data Compass: https://doi.org/10.17037/DATA.00001753.

## Ethics Statement

The study was approved by the London School of Hygiene and Tropical Medicine Observational research ethics committee (Ref: 21846). All data were fully anonymous and the study team had no means by which they could identify individual respondents. All participants provided consent to participate in the study by ticking a box on the survey web-form. All questions in the survey were optional (excepting age and number of people in the household), meaning that participants could skip questions if they did not want to divulge specific data.

## Author Contributions

NR, SL, and CR conceptualized the study. NR, HB, LE, RE, SL, and CR designed the survey. HB and CR deployed the survey and curated the data. NR, NW, and CR performed the analysis. NR and CR wrote the manuscript. All authors reviewed the manuscript. All authors contributed to the article and approved the submitted version.

## Conflict of Interest

The authors declare that the research was conducted in the absence of any commercial or financial relationships that could be construed as a potential conflict of interest.

## Footnotes

^1^Available online at: https://www.who.int/news-room/detail/30-01-2020-statement-on-the-second-meeting-of-the-international-health-regulations-(2005)-emergency-committee-regarding-the-outbreak-of-novel-coronavirus-(2019-ncov).

^2^World Health Organization. Coronavirus disease 2019 (COVID-19) Situation Report – 101. Available online at: https://www.who.int/emergencies/diseases/novel-coronavirus-2019/situation-reports.

^3^Covid-19: Home based exercise activities could help during self-isolation. The BMJ. 2020. Available online at: https://blogs.bmj.com/bmj/2020/03/16/covid-19-home-based-exercise-activities-could-help-during-self-isolation/.

^4^Health Survey for England 2016 Physical activity in adults Available online at: healthsurvey.hscic.gov.uk/media/63730/HSE16-Adult-phy-act.pdf.
