# Supplementary material for: Behavioral Change Towards Reduced Intensity Physical Activity Is Disproportionately Prevalent Among Adults With Serious Health Issues or Self-Perception of High Risk During the UK COVID-19 Lockdown
Source: Front Public Health. 2020 Sep 30;8:575091. doi: 10.3389/fpubh.2020.575091 (PMC7554527; doi:10.3389/fpubh.2020.575091)
Supplement: Supplementary file 1 [file Table_1.DOCX]

Behavioural change towards reduced intensity physical activity is disproportionately prevalent among adults with serious health issues or self-perception of high risk during the UK COVID-19 lockdown.

### **Supplementary Data**

**Nina Trivedy Rogers^2^, Naomi Waterlow^1^, Hannah Brindle^1^, Luisa Enria^3^, Rosalind M Eggo^1^, Shelley Lees^1^ & Chrissy h Roberts^1*^**

^1^ London School of Hygiene and Tropical Medicine, London WC1E 7HT, UK

^2^ UCL Research Department of Epidemiology & Public Health, London WC1E 7HB, UK

^3^ University of Bath, Department of Social & Policy Sciences, Bath BA2 7AY

*** Correspondence:**Corresponding Author
[Chrissy.Roberts@lshtm.ac.uk](mailto:Chrissy.Roberts@lshtm.ac.uk)

Supplementary Table S1 : Correlation between previous doctor diagnosis with lung disease and mention of asthma in the chronic disease diagnosis corpus

|  | No Mention Asthma | Mentioned Asthma | NA | Total |
| --- | --- | --- | --- | --- |
| No Lung Disease | 6304 (91.2%) | 573 (8.3%) | 34 (0.5%) | 6911 (75.2%) |
| Lung Disease | 281 (42.2%) | 377 (56.6%) | 8 (1.2%) | 666 ( 7.2%) |
| NA | 1397 (86.6%) | 187 (11.6%) | 29 (1.8%) | 1613 (17.%) |
| Total | 7982 (86.9%) | 1137 (12.4%) | 71 (0.8%) | 9190 (100.0%) |
| Pearson’s X-squared = 1345, df = 9, p-value < 2.2e-16  Percentages and marginal counts include NAs although the X-squared test omitted use of NAs as explicit factors | | | | |

##### Supplementary Table S2 : Data filtering for analysis.

| Filter | n | Count |
| --- | --- | --- |
|  |  | 9456 |
| Under 20 years | 67 | 9389 |
| No baseline PA Data | 146 | 9243 |
| No covid PA Data | 53 | 9190 |
| n indicates number of participants removed in each filtering step  Count is a running count of participants retained for analysis | | |

##### Supplementary Table S3 : Data used in multinomial log-linear model

|  |  | PA Intensity change during lockdown | | | |
| --- | --- | --- | --- | --- | --- |
| Variable | Group | Same  (N=5872) | Less  (N=2302) | More  (N=1016) | Total^1^  (N=9190) |
| PA intensity prior to lockdown  n (%) | None | 172 (59.7%) | 0 (0.0%) | 116 (40.3%) | 288 (3.1%) |
|  | Mild | 1999 (64.3%) | 390 (12.5%) | 722 (23.2%) | 3111 (33.9%) |
|  | Moderate | 2587 (66.8%) | 1109 (28.6%) | 178 (4.6%) | 3874 (42.%) |
|  | Vigorous | 1114 (58.1%) | 803 (41.9%) | 0 (0.0%) | 1917 (20.9%) |
| PA intensity during lockdown  n (%) | None | 172 (23.2%) | 568 (76.8%) | 0 (0.0%) | 740 ( 8.1%) |
|  | Mild | 1999 (60.5%) | 1229 (37.2%) | 76 (2.3%) | 3304 (36.0%) |
|  | Moderate | 2587 (68.7%) | 505 (13.4%) | 672 (17.9%) | 3764 (41.0%) |
|  | Vigorous | 1114 (80.6%) | 0 (0.0%) | 268 (19.4%) | 1382 (15.0%) |
| Age  n (%) | 20-34 | 317 (51.3%) | 204 (33.0%) | 97 (15.7%) | 618 (6.7%) |
|  | 35-54 | 1940 (59.3%) | 920 (28.1%) | 414 (12.6%) | 3274 (35.6%) |
|  | 55-69 | 2858 (67.5%) | 942 (22.2%) | 436 (10.3%) | 4236 (46.1%) |
|  | 70+ | 757 (71.3%) | 236 (22.2%) | 69 (6.5%) | 1062 (11.6%) |
| Gender  n (%) | Female | 4502 (63.0%) | 1815 (25.4%) | 826 (11.6%) | 7143 (77.7%) |
|  | Male | 1285 (67.1%) | 450 (23.5%) | 179 (9.4%) | 1914 (20.8%) |
|  | All other genders | 43 (58.9%) | 21 (28.8%) | 9 (12.3%) | 73 ( 0.8%) |
|  | Imputed | 42 (70%) | 16 (26.7%) | 2 (3.3%) | 60 ( 0.7%) |
| Living alone  n (%) | No | 4915 (64.2%) | 1880 (24.5%) | 866 (11.3%) | 7661 (83.4%) |
|  | Yes | 957 (62.6%) | 422 (27.6%) | 150 (9.8%) | 1529 (16.6%) |
| Education  n (%) | GCSE/O-level | 595 (64.1%) | 232 (25.0%) | 101 (10.9%) | 928 (10.1%) |
|  | A level/Highers | 1630 (64.6%) | 632 (25.0%) | 261 (10.3%) | 2523 (27.5%) |
|  | Degree | 3481 (63.3%) | 1380 (25.1%) | 641 (11.7%) | 5502 (59.8%) |
|  | Imputed | 166 (70%) | 58 (24.5%) | 13 (5.5%) | 237 ( 2.6%) |
| Access to a garden  n (%) | Yes | 5423 (65.1%) | 2000 (24.0%) | 910 (10.9%) | 8333 (90.6%) |
|  | No | 433 (51.9%) | 298 (35.7%) | 103 (12.4%) | 834 (9.1%) |
|  | Imputed | 16 (69.6%) | 4 (17.4%) | 3 (13%) | 23 ( 0.3%) |
| School aged children  n (%) | No | 4668 (65.1%) | 1756 (24.5%) | 744 (10.4%) | 7168 (78.0%) |
|  | Yes | 1155 (59.1%) | 533 (27.3%) | 267 (13.7%) | 1955 (21.3%) |
|  | Imputed | 49 (73.1%) | 13 (19.4%) | 5 (7.5%) | 67 ( 0.7%) |
| Household Income  n (%) | Less than £15,000 | 664 (63.9%) | 262 (25.2%) | 113 (10.9%) | 1039 (11.3%) |
|  | £15,000 - £24,999 | 968 (64.4%) | 381 (25.3%) | 154 (10.2%) | 1503 (16.4%) |
|  | £25,000 - £39,999 | 1163 (63.8%) | 438 (24.0%) | 223 (12.2%) | 1824 (19.8%) |
|  | £40,000 - £59,999 | 1058 (63.6%) | 443 (26.6%) | 162 (9.7%) | 1663 (18.1%) |
|  | £60,000 - £99,999 | 836 (62.9%) | 332 (25.0%) | 162 (12.2%) | 1330 (14.5%) |
|  | More than £100,000 | 382 (61.9%) | 151 (24.5%) | 84 (13.6%) | 617 ( 6.7%) |
|  | Imputed | 801 (66%) | 295 (24.3%) | 118 (9.7%) | 1214 (13.2%) |
| Disability (ADL)  n (%) | No | 5613 (63.8%) | 2189 (24.9%) | 990 (11.3%) | 8792 (95.7%) |
|  | Yes | 259 (65.1%) | 113 (28.4%) | 26 (6.5%) | 398 (4.3%) |
| Depression  n (%) | No | 5362 (65.3%) | 1942 (23.6%) | 912 (11.1%) | 8216 (89.4%) |
|  | Yes | 465 (51.4%) | 342 (37.8%) | 98 (10.8%) | 905 ( 9.8%) |
|  | Imputed | 45 (65.2%) | 18 (26.1%) | 6 (8.7%) | 69 ( 0.8%) |
| Diabetes type I  n (%) | No | 4683 (63.0%) | 1914 (25.7%) | 840 (11.3%) | 7437 (80.9%) |
|  | Yes | 57 (69.5%) | 17 (20.7%) | 8 (9.8%) | 82 ( 0.9%) |
|  | Imputed | 1132 (67.7%) | 371 (22.2%) | 168 (10.1%) | 1671 (18.2%) |
| Diabetes type II  n (%) | No | 4530 (63.1%) | 1839 (25.6%) | 813 (11.3%) | 7182 (78.2%) |
|  | Yes | 392 (67.7%) | 130 (22.5%) | 57 (9.8%) | 579 ( 6.3%) |
|  | Imputed | 950 (66.5%) | 333 (23.3%) | 146 (10.2%) | 1429 (15.5%) |
| Lung Disease  n (%) | No | 4026 (63.5%) | 1583 (25.0%) | 729 (11.5%) | 6338 (69.0%) |
|  | Yes | 876 (61.4%) | 403 (28.3%) | 147 (10.3%) | 1426 (15.5%) |
|  | Imputed | 970 (68%) | 316 (22.2%) | 140 (9.8%) | 1426 (15.5%) |
| Cancer  n (%) | No | 4332 (63.0%) | 1758 (25.6%) | 790 (11.5%) | 6880 (74.9%) |
|  | Yes | 474 (67.7%) | 171 (24.4%) | 55 (7.9%) | 700 ( 7.6%) |
|  | Imputed | 1066 (66.2%) | 373 (23.2%) | 171 (10.6%) | 1610 (17.5%) |
| Stroke  n (%) | No | 4544 (63.1%) | 1838 (25.5%) | 814 (11.3%) | 7196 (78.3%) |
|  | Yes | 94 (61.0%) | 45 (29.2%) | 15 (9.7%) | 154 ( 1.7%) |
|  | Imputed | 1234 (67.0%) | 419 (22.8%) | 187 (10.2%) | 1840 (20.0%) |
| Heart disease  n (%) | No | 4461 (63.1%) | 1807 (25.6%) | 803 (11.4%) | 7071 (76.9%) |
|  | Yes | 273 (65.8%) | 105 (25.3%) | 37 (8.9%) | 415 ( 4.5%) |
|  | Imputed | 1138 (66.8%) | 390 (22.9%) | 176 (10.3%) | 1704 (18.5%) |
| Hypertension  n (%) | No | 3884 (63.2%) | 1569 (25.5%) | 688 (11.2%) | 6141 (66.8%) |
|  | Yes | 1414 (65.2%) | 519 (23.9%) | 237 (10.9%) | 2170 (23.6%) |
|  | Imputed | 574 (65.3%) | 214 (24.3%) | 91 (10.4%) | 879 ( 9.6%) |
| Obesity  n (%) | No | 4013 (63.7%) | 1613 (25.6%) | 678 (10.8%) | 6304 (68.6%) |
|  | Yes | 941 (61.4%) | 381 (24.9%) | 211 (13.8%) | 1533 (16.7%) |
|  | Imputed | 918 (67.8%) | 308 (22.8%) | 127 (9.4%) | 1353 (14.7%) |
| ^2^ Total shows percentage of each class in the total sample of 9190 participants.  Rows marked ‘Imputed’ show the number of survey responses in which imputation was applied to fill missing data | | | | | |

Supplementary Table S4 : Topic Perspectives (Selected exemplar quotes) from text corpus on coping behaviour during lockdown.

| **Topic T1 : Perceptions of risk, working or living in risk environments, already had COVID-19** |
| --- |
| I looked at what was happening in Italy and their criteria for patients accessing ventilators. During the height of the pandemic I do not believe I would be given a ventilator. I therefore got some activities (mainly craft) to last at least 12 weeks. I self isolated one week before the government issued lockdown measures |
| Trying to recover from covid 19. My symptoms started on the 23 March as well and I am still very sick and in bed. |
| My husband has had CV and is a kidney transplant patient. He is recovering after being hospitalised with a mild virus of CV we are now in week 3 post initial infection |
| I am now working from home - only two mornings a week which were my previous hours. I still walk my dogs first thing in the morning. Only two things have really changed for me, the first and most insignificant is that I do the shopping, which was previously done by my husband, but as he's diabetic with a heart condition I have taken over, AND most importantly I used to see my grandchildren 4 or 5 times a week - 2 of those days I was looking after them. Not seeing them is the worst thing about lockdown for me. It's heartbreaking |
| I am a cancer patient recovering from surgery. I was off work at the end of December. I am used to being at home. |
| That was too late for me already got the virus from a patient as an nhs worker . I have been in bed ill for 3 weeks upto now with no proper care at all . Very shameful of this government not to have acted sooner |
| It wasn't a complete lockdown. Italy locked down and you needed a pass to go shopping in the UK you can mass purchase 7 days a week. I was originally on a shift rota for work but my dad is extremely high risk so I am shielding. I am doing everything I can to work from home and help those at work however small the task to alleviate pressure on them. I exercise to help. I have started doing puzzles and reducing my news outlet to once per day. |
| I made the decision to move to my parents home to care for them during lock down. They are 94. They woukd not have coped without me being here. It is hard being removed from my own home. |
| "I live and work on a small farm. My wife and I both have asthma. Our lives have changed very little: all the animals still need the same care every day. Our fledgling free range pedigree pork business has only just begun, so we are ineligible for any grants or help. We used not to go out very often in any case - perhaps two or three times a month for a trip to the beach, a pub lunch or afternoon tea." |
| Work in NHS, which shapes many of my answers (eg work colleagues and work newsletter v trustworthy). So I still go to work, have childcare arrangements as partner also NHS, visit elderly parents to restock them. Chat online or over phone. Do exercise. Work expands into days off though |
| **Topic T2 : Adherence to guidelines / Social distancing** |
| I tried to help others as much as possible while respecting my own needs and fears. I don't go out, I avoid public spaces and transports, I wear protective gloves and face masks when I'm outside and in common areas. And keep social distancing as well as wash my hands regularly, when it's the case that I need to wash them. |
| Getting to know loved ones better and using time, a precious commodity. |
| Following government lockdown advice. |
| Staying inside and using any delivery services I can for essentials and food. Buying gloves and masks to stay safe when I do have to go out. Keeping more than 2m away from everyone. Only go out for food shopping or a brief walk. Haven't met face to face with any friends, family or neighbours, instead use social media, texting or calls. I have sever mental health problems and all my appointments are cancelled. I struggle to go to the shop as social distancing is not being adhered to. I have been more social media and messaging, I have found medical leaders and corvid19 experts to follow on Twitter for my news. I don't watch the news on tv, read it online or listen to it, as I find it unreliable and also very depressing. |
| Social distancing |
| We made the decision to self isolate 3 days before the lockdown. People weren't following social distancing advice. The shops and supermarkets are getting it right now but they should have organised hand sanitisers and methods of social distancing right from the outset. |
| Following the rules |
| Using social media and Zoom etc. to communicate with people and pass the time. |
| Avoid contact with people. Not taking any pubblic transport. Using face masks when I go to buy food |
| following government advice |
| **Topic T3 : Activities around the house** |
| Arts and crafts, Sewing, Knitting, Gardening, Listening to music, Listening to Christian radio, Watching television |
| Sewing, knitting, baking, gardening, reading, watching tv, listening to the radio. |
| Jigsaw puzzles, reading, fixing things in the house, cooking, gardening, decorating, cleaning, organising cupboards, decluttering |
| Walking, cycling, gardening, sewing, decorating, housework, reading, FaceTiming family. Cooking. |
| Walking, gardening, reading, texting,phoning, sewing , knitting, watching television, cooking. |
| Reading, watching TV, watching films, cooking, tidying cupboards, gardening, beauty treatments, music |
| Decorating, gardening, cleaning, sorting garage, wardrobes, cupboards , exercising , watching tv and reading, cooking |
| Gardening, listening to music, watching films, doing puzzles, reading |
| Gardening, dog walking, reading, painting, tidying house, sorting cupboards |
| Decorating, gardening, walking, sorting cupboards, reading, cooking, watching films, playing games on line. |
| **Topic T4 : Social media / Online activities** |
| Behaving in an adult manner, informed by my scientific background. Engaging at a safe distance with my immediate neighbours. Connecting with in-laws via WhatsApp and Facebook. Speaking regularly with my daughter. And spoiling my pets rotten! |
| Joined support group - providing IT assistance, gardening, online grocery shopping for vulnerable neighbours |
| Lots more messages eg WhatsApp and video calls with family and groups I'm in.  Bought an electric bicycle so I can cycle again- and with my husband! |
| Walking. Joining online support groups. Connecting on zoom, by texts, phone etc |
| Keeping in touch with friends via phone and video chat, singing workshops on zoom, local walks |
| Weekly flute lesson now done via Skype. Local churches have set up a WhatsApp group which helps and supports us. We share music, poems, prayers, jokes, photographs etc. Watched our Rector‚Äôs streamed Easter services. Catching up with television programmes. |
| Video calls with friends & family. Street WhatsApp group so neighbours can help one and other. |
| Involved in producing magazine for local community with information and interesting articles. Providing support for beneficiaries in national charity I am trustee with esp when food difficult to locate at fair price. Knitting for a young friend. Reading. Cooking. Gardening. Hosting zoom mtgs for family here and in NZ. Hosting zoom mtgs for groups in village |
| I have continued with my Zumba and Pilates via Zoom. I have had virtual meetings with friends. I‚Äôm have emailed. have talked to my family on the phone and used FaceTime to speak to my children and grandchildren. I have painted rooms and gardened plus reading doing crosswords and puzzles. |
| Praying, Being involved in streaming church services, Calling people I know who are alone, Gardening |
| **Topic T5 : Staying home & only leaving house to shop** |
| Not leaving the house except a short walk, for a weekly food shop and to deliver food to elderly parents. |
| Staying home and relying on my daughter for food shopping and twice on a home delivery from Waitrose |
| I have been staying at home since then. I only left my house for food once a week (for 20 minutes). |
| Staying in! Only going out for essential shopping around once a week. |
| mainly making sure I am 2 meters away from everybody except my wife , washing my hands frequently and sanitizing all products delivered to our house or purchased in a shop. |
| Trying to source food without going out. Unfortunately no delivery slots left - so forced to go out to buy food. |
| Staying home only going to the shops when necessary |
| Self isolating and staying at home. |
| Stayed within the house without going outside at all apart from in the garden since we returned from holiday on 19 March. |
| Haven't had any visitors, haven't left the house except for shopping, have closed our b&b, have only been shopping when we ran out of milk or dog food. |
| **Topic T6 : Positivity / Health / Exercise** |
| Sticking to a routine, keeping fit, eating well, trying to stay positive |
| Exercising, getting fresh air, mindfulness, trying to eat & sleep properly, keeping in touch with friends & colleagues. |
| Keeping in touch with friends and family, keeping a routine and doing creative things to keep entertained. |
| Keeping in touch with friends and family, exercising, trying to keep routine. |
| Keeping in contact with family / friends, Trying to have some structure / routine to each day, Doing some exercises each day |
| Ensuring we have our daily exercise. Have activities to do each day. Speak to family and friends each day. Try and remain positive. |
| Keeping a routine, Keeping in touch with friends&family( non contact type), Keeping busy |
| constant communication with family, friends. daily routine- prayers, exercise, healthy diet, hobbies. |
| Staying in more regular contact with family and friends in the UK and abroad; trying to establish a schedule every day. Making sure we eat well, get outside every day, and take time not staring at a screen |
| Keeping a routine, VITAL getting up dressed and showered at similar times, and most of all, go out for walk an hour before sunset, everyday (something to look forward to in the day) |
| **Topic T7 : Mental health / Anxiety / Nonchalance** |
| I'm bipolar so this pandemic isn't scaring me. I've lived my whole life in a scary place, and this is nothing compared to that. I was prepared as I knew last year something would mess up causing this. So I've felt fine. I'm organised so I've managed to help a few other people with shopping and also check in on them as now they are anxious. |
| I am not copeing well. I was just emerging from a depressive episode that had lasted six months. Now I'm feeling much worse and would rather die than live like this. So many of the events that I had booked, which were what was giving me a reason to look forward to and keep going, have now been cancelled. There's nothing to look forward to and being stuck in doors with no gym or friends to see is miserable. This life is not worth living. |
| Just carrying on as usual, except for spending more time trying to get groceries and other supplies using the internet or with help from other people.  A really big blow has been having to have my cat put to sleep 4 days ago when he lost the use of his back legs. He was my companion and he structured my days from waking me up most mornings. I have been very tearful since then and am not sure if and when I will get on a more even keel. For want of someone sympathetic to talk to I did phone Samaritans a couple of evenings ago and it was helpful, as I was feeling guilty about being so upset about a cat when lots of people deaths were also happening. The talk was helpful but I haven't got round to going out as advised - it always seems a bit of an effort, even in good times, but I do usually go out to the village once a week to get a few groceries I can carry. Now I am a bit afraid to go to the village shop because of the virus and have no car so can't go to a supermarket,and can't get lifts because of social distancing. I don't see the point of just walking without some purpose at the end of it. In the past I would usually garden but my knees hinder me now and I quickly get tired.   I am normally dependent on Tesco deliveries and miss them (and even brief chats with the delivery men) very much. I cannot see deliveries for people without priority resuming for months and this is a big worry. I can remember food being short during the 2nd World War but at least then you were entitled to certain basic supplies. That is different from now and I somehow can never bring myself to fight for food - I would prefer to go hungry, but then I have never had to provide for children or other dependants.   My main hobby is family history, which takes me out of myself in trying to solve problems, but I don't seem to be able to get down to it at present as my mind wanders off and also there are more things to organise, like getting food. I've lost weight with not being able to get what I usually eat.  Perhaps the worst thing has been not being able to get hugs since the cat died, though people, including the vet, have wanted to give me them. My nearest family members are 120 miles away and most would be sympathetic, but I do not know when I will see any of them again as we do not meet regularly. |
| Trying to control anxiety. Trying not to feel guilty because of lack of motivation to do things I feel I should do and have time to do: social media shows many energetic motivated people and it's easy for that to make me feel worse. I am fortunate: I am not short of money, I have a garden and live in an area of outstanding natural beauty and this makes me feel guilty for being what I see as pathetic. It really helps if I am asked to do something for a vulnerable person via our village support network. |
| Not coping at all. Mental health has been really affected |
| I am alone anyway. So in a perverse way, the fact that others now have to live simply and quietly by themselves is a strange comfort. Been involved in NHS all my adult life (ex husband a Doctor) In the past, was sad that I had wasted my life caring so much. Now feel proud to have carried that torch of humanity, and see it blaze in this time of uncertainty. It is all that really counts in a life lived. |
| i have not been coping I feel that the health services have been deliberately run down for a long time I am considered extremely high risk for health reasons. My immune system has to stay weak for the rest of my life. It it just a matter of time before I catch the virus, and then it will be difficult for me to survive it, so I am trying not to have any false hope we are being left to die and the people responsible are gaining in popularity |
| I'm used to isolation as I have a chronic pain condition. It's everyone else who isn't coping haha! |
| I live with a shielded person so I have not been out either as he is terrified of getting it as he sees it as a death sentence because of the severity of his health problems |
| I have 6 yr old grandson living with me, he is a difficult child, so this makes coping more difficult. I would do better coping on my own. |
| **Topic T8 : Balancing work, family & caring** |
| I'm a key worker so have been working, when at home spending time with my kids and home improvements |
| I've been working as a key worker. Looking after my husband and daughter at home with covid 19. |
| I am a key worker so still working but spending more time with my daughter |
| I am a key worker so still working. Also a carer for terminally ill husband so spend a lot of time cleaning the home |
| I have enjoyed spending time with my husband and children |
| I am a key worker so still have to go out to work to look after elderly, frail people. Spending some quality time with my son. |
| Spending a lot of time in our garden. I am working full time as is my husband and we have a 10 yr old at home - we are also delivering food to my parents so tbh that occupies our mental space. |
| Working from home, spending time with my daughter. |
| I'm still working full time as I'm bank staff and classed as essential |
| Working full time and looking after my boys. Couch to 5km |
| **Topic T9 : Exercise** |
| Online yoga, fitness, outdoor walks, watching science documentaries, talking to family, messaging friends. Meditation apps |
| Exercise classes, going for more frequent walks/short cycle rides, video chats with friends/colleagues, cooking. |
| I'm a tutor. Been frantically trying to learn online teaching methods, messaging family and friends, doing lots of exercise - running and cycling. |
| Exercise, speaking with friends and family, games |
| Online yoga and fitness classes to replace the classes I did before |
| Regular contact with friends and family including video chats, virtual quizes, online classes (baby & toddler), online exercise classes. |
| Video games, exercise and hobbies |
| Exercising more, running Instagram live quizzes for family and friends, playing consoles |
| Cycling, doing online courses, applying for voluntery roles |
| Baking, Exercise bike, Walking dogs (once a day), Talking to friends and family, Online courses |
| **Topic T10 : Gardening & outdoor life** |
| Staying in does not bother me...I have a small dog which I walk in the morning a d my husband walks p.m. I have a lovely small garden to sit in and have hobbies a d enjoy reading |
| I have a huge garden and have been enjoying the better weather tending the garden, preparing ground and planting seeds. There is space to walk the dog. I get the weekend newspapers locally and tune in to the 5 pm briefing every night. |
| Moved from flat to house. Doing lots of DIY. Built greenhouse, and growing veg. Making bread... etc |
| i'm lucky in that I have a fairly large garden including small allotment. Reading more. |
| More gardening, thankfully I am lucky to have a back garden. Buying bulbs, plants, getting to grips with a backlog of weeding and garden maintenance. More cooking. Preparing a large quantity of meals and freezing individual portions. Much more sleeping. |
| I'm making and coordinating scrubs for GP practices for their Covid hub. I have a large garden so growing more plants and vegetables, cooking more and being creative with my veg and fruit box, face timing my family and friends |
| Walk once a day, sit in the garden , sit in the hot tub with beer and netflix |
| Walking in the countryside (we are lucky where we live) with our dog, skipping with a rope and doing Pilates to keep fit, gardening - lots - (again, we are lucky), growing veg and plants in my greenhouse, lots more cleaning obviously, and watching TV and knitting in the evenings. If weather bad, playing the piano and ukulele. Basically I am active all day and inactive in the evenings. |
| Walkng the dog each day approximately 3 miles in beautiful countryside on our doorstep. This has kept us sane. We have a garden and enjoy lots of pastimes which keep us busy. We are extremely lucky. We have each other. |
| Going outdoors (garden, allotment, or walk / bike ride (at a safe distance fro others) every day. Keeping busy (wildlife recording in garden, trying to sell wife's newly published book online, sowing and planting at allotment). These are things I would do anyway, to keep myself sane. |
| **Note : The 10 quotes presented for each topic were selected from a list of 40 quotes per topic that STM created. The authors chose to include quotes in this selection which they felt best represented the distinct characteristic themes of the different topics. The quotes are presented in the original form, which may include spelling and grammatical errors, as well as the use of language that some may find offensive. These quotes are perspectives of the study participants and do not reflect the opinions of the study authors.** |

##### Supplementary Figure S1 : Topic Proportions (a) and word frequency cloud (b) for STM of chronic disease diagnosis free-text data. Asthma was the most frequently mentioned word and asthma related topics was prevalent in the corpus of text.
